# Dogs suppress a pivotal function in the food webs of sandy beaches

**DOI:** 10.1038/s41598-022-18194-9

**Published:** 2022-08-18

**Authors:** Brooke Maslo, Robert Kwait, Christian Crosby, Price Holman, Isabelle Zoccolo, Kathleen Kerwin, Todd Pover, Thomas A. Schlacher

**Affiliations:** 1 Department of Ecology, Evolution and Natural Resources, Rutgers, The State University of New Jersey, New Brunswick, NJ USA; 2 Conserve Wildlife Foundation of New Jersey, Princeton, NJ USA; 3 School of Science and Engineering, University of the Sunshine Coast, Maroochydore, Australia

**Keywords:** Biodiversity, Community ecology, Ecosystem services, Invasive species, Ecology

## Abstract

Domestic dogs are the most abundant carnivore globally and have demonstrable negative impacts to wildlife; yet, little evidence regarding their functional roles in natural food webs exists. Adding dogs to food webs may result in a net loss (via suppression of naturally occurring species), net gain (via mesopredator release), or no change (via functional replacement) to ecosystem function. Scavenging is a pivotal function in ecosystems, particularly those that are energetically supported by carrion. Dogs also scavenge on animal carcasses, but whether scavenging by dogs influences the structural and functional properties of food webs remains unclear. Here we used camera traps baited with carrion to test the effect of dogs on the composition and diversity of the vertebrate scavenger guild, as well as carrion detection and consumption rates. We conducted this work in sandy beach ecosystems, which rely on the import of marine organic matter (i.e. stranding of dead marine animals). Diversity of the scavenger community was similar on beaches without dogs. Dogs increased the time it took for carcasses to be detected and decreased the proportion of carrion consumed. This ‘dog suppression effect’ on scavenging was stronger for nocturnal mammalian scavengers, presumably being driven by indirect trait-mediated effects, which raises further questions about the broader ecological consequences of domestic dogs in natural systems.

## Introduction

Dogs (*Canis familiaris*) are the most abundant carnivore globally^1^. They have been shown to disrupt wildlife communities across several ecosystems and are considered an increasingly significant driver of vertebrate population declines^2,3^. Although dogs are typically considered sustained by human-supplied food^4,5^, they can exert significant predation pressure across a range of taxonomic groups and life stages^6,7^. Interestingly, dogs do not always consume the animals they kill^8,9^. Where direct killing does not occur, dog pursuit or harassment of prey species can trigger both immediate and downstream deleterious effects on prey fitness^7,10,11^. Finally, dogs can assume the role of an apex predator in many ecosystems, altering trophic structure of the existing community or displacing native carnivores via intraguild competition^4,12,13^. The majority of studies on the interactions between dogs and wildlife focus on free-ranging dogs^12^, but even leashed dogs can trigger anti-predator or avoidance behaviors in prey or intraguild species^14,15^.

Despite their global ubiquity, little is known about the functional role of domestic dogs in natural ecosystems. Most dog-focused studies report on direct impacts on behavior and populations of wildlife^3,16,17^. In some cases, effects on ecological processes (i.e. trophic cascades,^18^) are implied; however, explicit investigations into how those effects modulate ecosystem function are rare. Because dogs act as trophic regulators within ecological communities^4^, and can be considered an invasive species in many contexts^19^, their presence in an ecosystem likely influences biodiversity-based ecosystem function. Dog presence may reduce ecosystem functioning directly by lowering species richness of ecological communities^20,21^. Alternatively, dogs may increase biodiversity and ecosystem functioning through competitive or predatory release mechanisms^22^, whereby the suppression of resident consumers facilitates populations at lower trophic levels. Finally, dogs may have no net impact on ecosystem function if they fulfill the functional role of the resident species they displace^23^. As the human footprint continues to expand worldwide into previously undisturbed habitats, and simultaneously knowledge of the value of biodiversity to human health and well-being is growing^24,25^, there is an urgent need to understand and potentially mitigate the impacts of ‘man’s best friend’ on natural systems.

Ocean-exposed sandy beaches represent an ideal ecosystem in which to quantify the effect of dogs on ecosystem function. Sandy beaches are of immense economic and cultural importance, and human reliance on them has increased and diversified to an expansive list of commodities (i.e. fish and shellfish, minerals, base materials for construction;^26^). Beaches also provide an array of services, most notably those regulated by the physical environment (i.e. storm protection, water purification, human recreation;^27^. However, ocean beaches are also highly functioning ecosystems, largely due to the taxonomic diversity sustained by organic matter subsidies from adjacent terrestrial, marine and estuarine habitats^28,29^.

Because of their symbiotic relationship with humans^30,31^, owned dogs (leashed, or unleashed and accompanied by humans) are often present on ocean beaches^19,32^. Much attention has been paid to the impacts of dogs on survival and reproduction of threatened species, particularly shorebirds^19,33,34^. However, the presence of owned dogs may have quantifiable direct or indirect effects on the ecology of coastal wildlife communities and the function of beaches altogether.

Although dogs are typically considered active predators, they also scavenge when animal carcasses are encountered^35^. While scavenging behavior in free-ranging domestic dogs is clearly demonstrated, it remains unclear whether scavenging by owned dogs influences the structural and functional properties of food webs that are energetically supported by dead animals. Consequently, here we use a camera-trapping study to test how the presence of owned dogs in sandy-beach food webs affects the species composition and diversity of the vertebrate scavenger guild, and we quantify functional responses (e.g. carrion detection and consumption rates) associated with the presence of owned dogs in beach food webs.

## Results

### Composition of the scavenger guild

**Figure 1:**
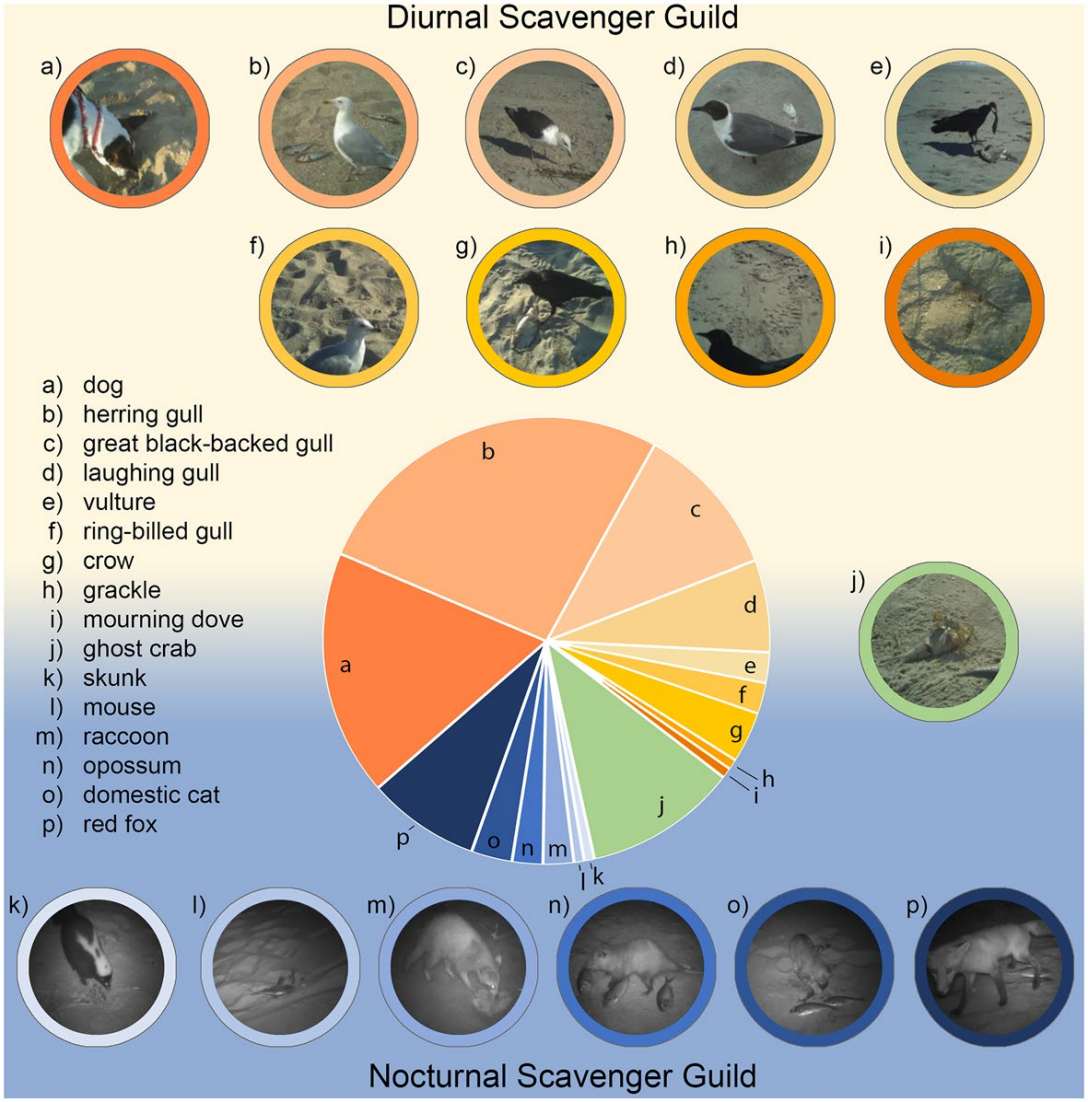
Scavenger species captured with remote infrared motion-triggered cameras on ocean-exposed beaches in New Jersey, USA. Pie chart indicates the proportion of camera locations at which each species was captured. Diurnal scavengers included (**a**) dogs (*Canis familiaris*), (**b**) herring gulls (*Larus argentatus*), (**c**) great black-backed gulls (*L. marinus*), (**d**) laughing gulls (*Leucophaeus atricilla*), (**e**) turkey vultures (*Cathartes aura*), (**f**) ring-billed gulls (*L. delawarensis*), (**g**) crows (*Corvus brachyrhynchos/ossifragus*), (**h**) common grackles (*Quiscalus quiscula*), and (**i**) mourning doves (*Zenaida macroura*). Ghost crabs (*Ocypode quadrata*) (**j**) were observed during both deployment periods. Nocturnal scavengers included (**k**) skunks (*Mephitis mephitis*), (**l**) white-footed mice (*Peromyscus leucopus*), (**m**) raccoons (*Procyon lotor*), (**n**) opossums (*Didelphis virginiana*), (**o**) domestic cats (*Felis sylvestris*), and (**p**) red foxes (*Vulpes vulpes*).

**Figure 2:**
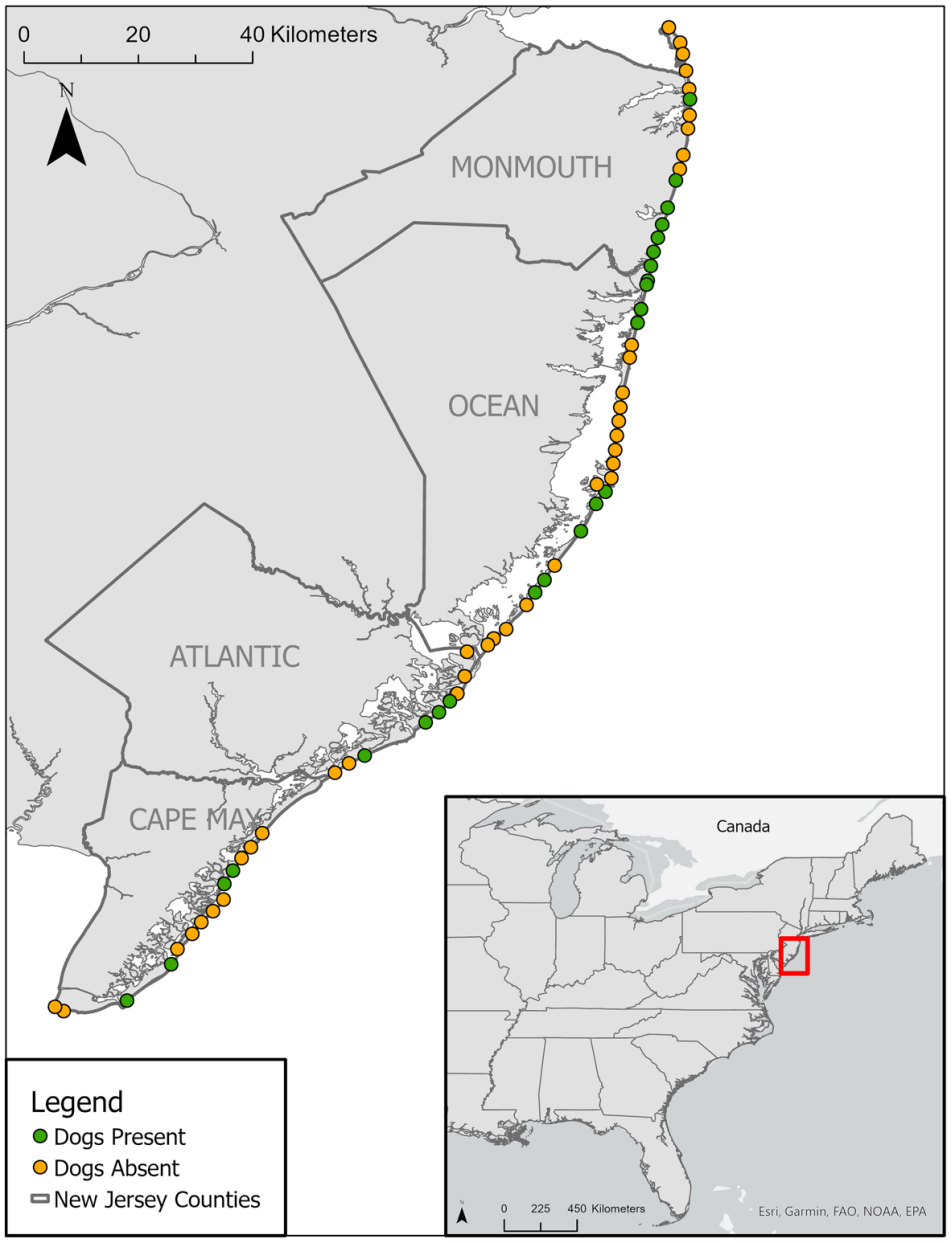
Camera deployment locations along ~ 250 km of the ocean-exposed coastline of New Jersey, USA. Green circles indicate camera locations where dogs were observed on cameras, and orange circles indicate camera locations where dogs were absent.

We detected 16 scavenger species in 5,636 camera trap images and 1,920 videos (Fig. 1; Table 1). The diurnal scavenging community was dominated by birds, including gulls, corvids, and vultures (the only obligate scavengers recorded); nocturnal scavengers were mostly mammals (Table 1). Atlantic ghost crabs (*Ocypode quadrata*) occurred at both day and night deployments at 24% of the camera locations. The most common bird scavengers were herring gulls (*Larus argentatus*), which we observed at 57% of camera locations. Great black-backed gulls (*L. marinus*) and laughing gulls (*Leucophaeus atricilla*) were also common, occurring at 24% and 14% of the camera locations, respectively. Ring-billed gulls were documented at only 3 camera stations (4%). Red foxes (*Vulpes vulpes*) were the most observed mammal (17% of camera locations), followed by domestic cats (*Felis sylvestris*, 6%), raccoons (*Procyon lotor,* 5%), and opossums (*Didelphis virginiana,* 5%). Resident mammals were only recorded at night. By contrast, owned dogs were only recorded during the day at 38% of camera sites (Fig. 2). Individual dogs were never observed at multiple, adjacent camera stations. Further, the dogs observed in this study were leashed, or unleashed and accompanied by a human companion. Free-ranging or feral dogs in our study region are rare and were not observed in our study.

**Table 1:** Frequency of occurrence of scavenger species at camera locations where dogs were and were not observed along ocean-exposed beaches in New Jersey, USA. Results of the SIMPER analysis are presented as the percent contribution to dissimilarity between observed communities with and without dogs.

Species	Occurrence (# of camera locations)		% Contribution to Dissimilarity
	Locations with Dogs (N = 24)	Locations Without Dogs (N = 39)	
**Diurnal Community (Average Dissimilarity = 76.60)**			
Herring gull	11	25	0.43
Great black-backed gull	4	11	0.21
Laughing gull	6	3	0.14
Crow	2	3	0.07
Turkey vulture	3	0	0.06
Ring-billed gull	1	2	0.04
Mourning dove	1	1	0.03
Grackle	0	1	0.01
**Nocturnal Community (Average Dissimilarity = 96.17)**			
Red fox	2	9	0.48
Cat	2	2	0.16
Raccoon	0	3	0.14
Opossum	1	2	0.12
Mouse	1	0	0.06
Striped skunk	0	1	0.05

### Altered scavenging on beaches with dogs

**Figure 3:**
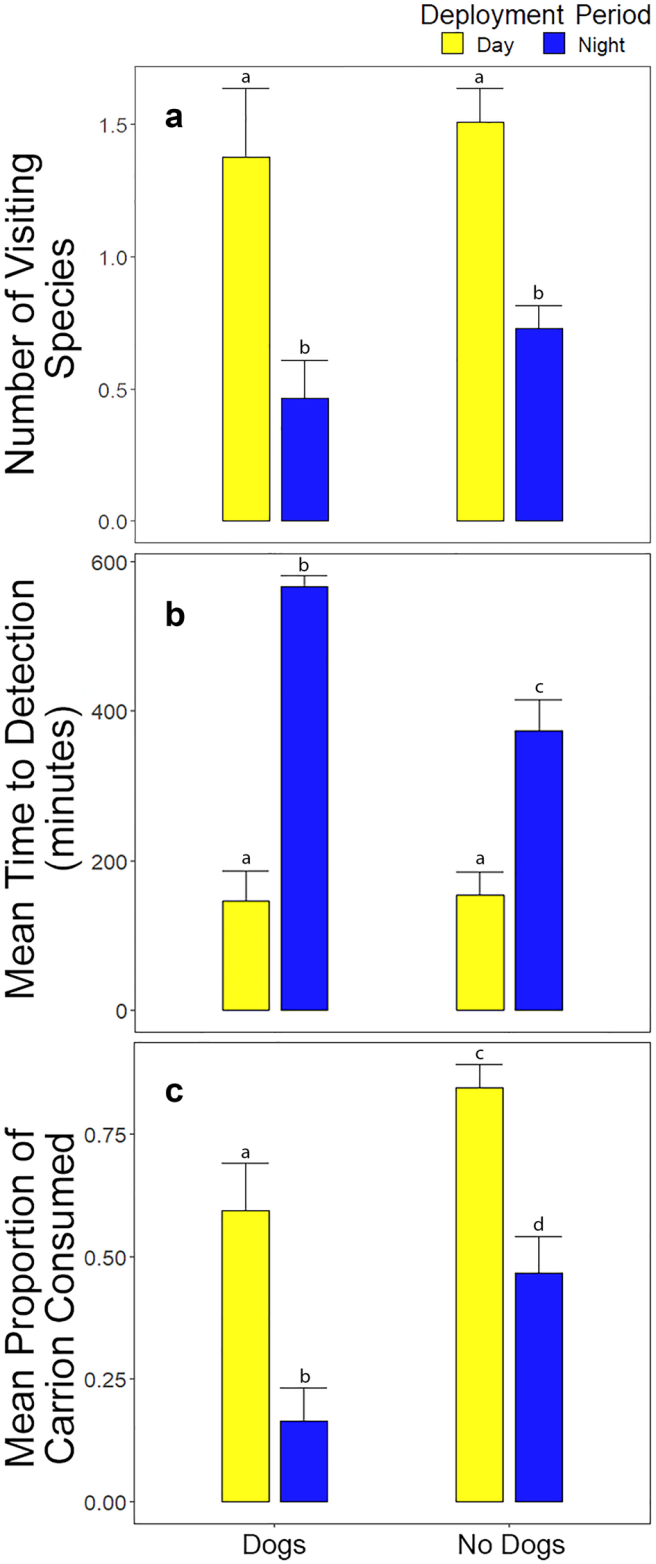
Differences in (**a**) mean number of visiting species; (**b**) time to first detection of carrion; and (**c**) proportion of carrion consumed at each camera location during diurnal (yellow) and nocturnal (blue) deployments (N = 63 deployments for each time period) and at sites with and without owned dogs. The height of the bar indicates the mean and the error bars represent the standard error for each category.

The scavenger community was significantly (ANOVA, *p* < 0.001; Fig. 3a) more species-rich during the day (1.83 ± 1.09 SD) than during the night (0.63 ± 0.62 SD), with deployment period (diurnal vs nocturnal) being the only significant factor for numbers of visiting species in our models (Tables 2 and 3). The mean number of mammal species detecting carrion in nocturnal carrion deployments was lower when dogs were present earlier that day (0.46 ± 0.14 SD) than when dogs were absent (0.73 ± 0.09 SD). The species composition of the scavenger assemblage differed significantly between sites with and without dogs (PERMANOVA, *p* = 0.018). Herring gulls contributed most to the dissimilarity of the diurnal scavenger guild between sites with and without dogs, (SIMPER % Contribution = 0.43). Red foxes contributed most to observed differences within the nocturnal scavenger guild (SIMPER % Contribution = 0.48). Raccoons, striped skunks, and grackles were not observed at sites where dogs were present, although overall detections of these species were low (Table 1). Ghost crabs, red foxes, and great black-backed gulls were observed ~ 80% less often on beaches with dogs.

**Table 2:** Top-ranked models describing the factors influencing the scavenger community and scavenging efficiency on ocean-exposed sandy beaches in New Jersey, USA. AIC_c_ is the Akaike’s information criterion corrected for small sample size; $\Delta$AIC_c_ is the difference between the AIC_c_ value for a given model and the top model; *w* is the Akaike weight.

Scavenging Metric	Model	AIC_c_	$\Delta$AIC_c_	*w*
# of visiting species	Deployment period + population density	302.2	0.0	0.23
	Deployment period	303.7	1.4	0.11
	Deployment period*(dist. To development + pop. Density)	303.9	1.6	0.10
	Dogs + deployment period + pop. Density	304.0	1.8	0.09
Time to detection	Deployment period + dogs	1405.7	0.0	0.32
	Deployment period*dogs	1406.5	0.8	0.22
Proportion of carrion consumed	Deployment period + dogs	144.4	0.0	0.39
	Deployment period + dogs + pop. Density	146.0	1.6	0.17
	Deployment period*dogs	146.1	1.7	0.16

**Table 3:** Model-averaged effect sizes for top predictors of scavenging efficiency on ocean-exposed beaches in New Jersey, USA. Models reporting a $\Delta$AIC_c_ < 2 were included in the analysis. Significant effects are in bold.

Scavenging Metric	Predictor(s)	Estimate (95% CI)	*P* value
# of visiting species time to carcass detection	**Deployment period (night)**	**−0.84 [−1.22, −0.47]**	**1.43e−05**
	**Deployment period (night)**	**2.75 [1.16, 4.33]**	**0.00079**
	Dogs	0.55 [−1.61, 2.71]	0.62
	Deployment period*dogs	0.62 [−1.67, 2.91]	0.60
Proportion of carrion consumed	**Deployment period (night)**	**−2.12 [−3.10, −1.14]**	**2.67e−05**
	**Dogs**	**−1.40 [−2.44, −0.36]**	**0.0088**
	Population density	−0.144 [-0.251, 0.183]	0.50
	Deployment period*dogs	0.58 [−1.06, 2.21]	0.52

Carrion went undetected in 32 of 126 carrion deployments. Where carrion was detected, the time to first detection ranged from 1 to 699 min (mean 194.1 ± 184.6 SD), with mean detection being ~ 80 min faster during the day. Detection times did not differ significantly among species during the night (*p* = 0.30); during the day, only crows and mourning doves had significantly shorter and longer detection times (*p* = 0.02), respectively. Dogs exhibited the widest range of carrion detection times, appearing on the camera between 8 – 699 min after carcass deployment (Supplemental Information–Figs. S1 and S2). Mammals took significantly longer to locate carrion at sites where dogs were present earlier in the day (ANOVA, df = 61, F = 7.781, *p* = 0.007; Fig. 3b). By contrast, the presence of dogs had no significant influence on carcass detection by birds during the day. When dogs were present at camera stations during the day, nocturnal scavengers that evening took approximately 34% longer to detect carrion [mean nocturnal carrion detection time (dogs present), 567 ± 15 SD minutes; (dogs absent) 373 ± 42 SD minutes]. Top models for time to carcass detection included the presence of dogs, deployment period, and the interaction of those predictors (Table 2). However, only deployment period was a significant predictor, indicating that scavengers took longer to find animal carcasses at night (effect size = 2.75, [1.16, 4.33]; Table 3).

Although dogs detected carrion at rates comparable with naturally occurring diurnal scavengers, dogs did not consume the fish carcasses we deployed. Dogs significantly depressed carrion consumption by resident scavengers (effect size = −1.40, 95%CI: −2.44, −0.36). This ‘suppression effect’ was stronger for nocturnal scavengers that consumed 3 × less carrion when dogs were at cameras sites that day (mean carrion consumption at night; dogs present 0.16 ± 0.07 SD; dogs absent 0.47 ± 0.07 SD; Fig. 3c).

## Discussion

On sandy shorelines, the concentration of carrion in a narrow strip coinciding with the land–ocean interface attracts consumer species from adjacent ecosystems^29^. Our camera trap records identified a scavenger guild consisting of coastal and marine birds that are known to feed on a broad range of food resources (e.g., gulls), obligate scavengers (e.g., turkey vultures), and generalists commonly found in several adjoining habitats (e.g., red foxes, corvids, raccoons). Dogs in our study did not affect overall scavenger diversity on ocean-exposed beaches, which was similar at sites with and without dogs. In fact, no hypothesized predictor (environmental or anthropogenic), except for deployment period, emerged as a driver of scavenger richness on beaches. This is expected given the adaptability of these species, which are habitat generalists that commonly exploit food and refuge resources in human-dominated landscapes^36–39^. Part of these consumers’ success is their ability to capitalize on subsidies provided by humans^36^. They also exploit seasonal, often predictable, pulses of food^40^, and they use a wide range of habitats^41^.

The vertebrate scavenger community in our study system showed complete temporal partitioning, with birds foraging exclusively during the day and mammals exclusively during the night. Dogs did not influence the number of bird scavengers detected by cameras. Similarly, dogs had no measurable effect on the time it took for carrion to be detected by birds during the day. Detection times varied significantly both within and among species, with diurnal scavengers generally taking less time to detect carcasses. All vertebrate diurnal scavengers in this study were birds, which have several adaptations that promote the efficient detection of carrion. Their keen vision and ability to fly allow birds to effectively search large areas more quickly than mammals^42^. Birds also transfer information to conspecifics on foraging site quality and location^43^, usually at roosts or colony locations^44^.

By contrast, we found considerably fewer species of mammals at carcass deployments on beaches with dogs. This ‘dog effect’ may operate by changing the behavior of individuals (through avoidance or shifts in temporal space use), by suppressing population sizes, or both. There were also significant differences in nocturnal carrion detection times at sites with and without dogs, with nocturnal scavengers taking much longer to detect carrion at sites where dogs were observed earlier that day. Despite the complete temporal partitioning of vertebrate scavengers in this study, nocturnal carnivores still appear to be affected by the presence of dogs in the habitat. Carnivores often avoid direct interactions with competitors by responding to olfactory signaling^45^; therefore, scent-marking by domestic dogs would be expected to initiate avoidance behavior in nocturnal scavenging mesopredators^46^. On multiple occasions, we observed dogs defecating and scent-marking at camera stations, supporting the hypothesis that the demonstrated absence of resident mammals is based on olfactory cues. Particularly on beaches, however, carrion is a critical trophic subsidy^47^. Rapid detection (and subsequent consumption) of carrion inputs can have important demographic benefits to consumers^48^. Animals may still engage in risky behavior and use habitats where strong competitors or predators are present to capitalize on rich carrion resources. These behaviors may come at a cost of increased search time, as scavengers must weigh the tradeoffs of predation risk and energy gain.

Previous research shows that scavengers detect and consume carrion quickly to maximize resource acquisition before it becomes unavailable through necrotic putrefaction^29,49^. Consistent with these studies, carrion in our experimental surveys was either completely consumed or not detected 83% of the time, indicating that if scavengers detected the carcasses, they consumed them. Yet, we observed reduced carrion consumption at study locations with dogs, regardless of deployment period. During the day, smaller scavengers likely avoid carrion resources in areas with dogs because of their propensity to chase, harass, and in some cases kill intraguild competitors^4^. At night, the effect of dogs on the proportion of carrion consumed was more pronounced, further underscoring the decoupled nature of interference competition by owned dogs on the scavenging community.

We posit two hypotheses, that are not mutually exclusive, to explain the apparent reduction in scavenger activity during nocturnal deployments. First, trait-mediated effects may carry over when olfactory cues left over from dogs, whether through scent marking or passive shedding, affect the behavior of some beach scavengers. The olfactory cues could result in complete avoidance behavior of mammalian scavengers and/or could reduce the efficiency of feeding at camera stations. In the latter case, scavengers may feed for shorter time periods or consume less of the carrion based on a fear response to the scent of a competitor or predator. In the case of a trait-mediated response, olfactory cues left by dogs would be short lasting and fast acting because the number of scavengers in the area would remain the same, but they would avoid areas with the scent of dogs^50^. Density-mediated effects may also be present, resulting in areas that tend to have a higher number of dogs regularly visiting the beach. If so, the higher abundance may reduce the local population of beach scavengers through direct or indirect means compared to areas with fewer dogs^51^. The lower population of beach scavengers would then result in longer detection times and lower consumption rates based solely on the reduced chance of a scavenger being in the vicinity. Density-mediated effects would likely be relatively slower acting and longer lasting because there would need to be a consistently higher number of dogs for a long enough time to reduce the local population of scavengers and recovery of the scavenger population would require immigration or reproduction.

It is also possible that another variable simultaneously affects the likelihood of seeing a dog at a camera station and the functional behavior of other beach scavengers. For example, if dogs are more likely to be seen at a camera station in urban areas and scavenger activity is different in urban areas than non-urban areas, the predictor ‘dog’ is confounded by urbanization. We included in our models several variables that index the broad type of land-use (natural vs. recreational) and the ‘human footprint’ near camera stations (e.g. human population density, distance to development). Supporting our conclusion of dogs impacting beach scavenging, our analyses showed that none of these other variables were significant predictors. By contrast, the variable ‘dog’ was a significant predictor of considerably longer time to carrion detection and lower proportion of carrion consumed.

Carrion detection times of dogs themselves was comparable to other diurnal species, but also exhibited the widest range. In addition, despite displaying a similar functional role as resident scavengers in detecting carrion, dogs in our study were never observed consuming carrion. These patterns were likely attributed to the human companions, who commonly walked their dogs either early morning (just after diurnal carrion deployment) or early evening (just before sunset). Human companions also likely prevented dogs from consuming carrion, as in some cases we observed owners pulling their dogs away from fish carcasses or removing them from their mouths. This suggests that wild or free-roaming domestic dogs probably assume a more pronounced functional role within the scavenger community, consuming carrion as a means of resource selection and survival^52^, and in turn limiting resource acquisition by resident scavengers through scramble competition. However, in our system dogs acted as inefficient members of the scavenging guild. Scavenging is a pivotal function in systems, especially in the food-webs of sandy beaches that are energetically supported by the import of marine organic matter, including the stranding of dead marine animals^53^. Rapid and efficient carrion removal also reduces pathogen loads^54^, thereby lowering the risk of wildlife disease and spillovers into human populations^55^. Therefore, the depressed rates of carcass removal on ocean beaches due to the presence of dogs raises the question of broader ecological consequences.

From a management perspective, owned dogs are typically treated as significant threats to other wildlife, especially species of conservation concern. Dogs have adverse impacts on many species of migratory and nesting shorebirds and marine turtles, among others, ranging from general disturbance to lowered fitness^56^. Furthermore, dogs can influence site distribution and occupancy of these species across the beach habitat and within coastal ecosystems. As a result, most conservation efforts for imperiled beach species include strategies to minimize or control dog activities. Most conservation areas prohibit dogs (both leashed or unleashed) during breeding seasons, or they fully close the beach to public access in critical sites. However, if the presence of dogs, as this study suggests, reduces the presence of other scavenger species on ocean beaches, it may have implications for how to best manage or utilize dogs in relation to at-risk species. Many of the scavenger species identified in this study, especially the nocturnal mammals, are also the focus of lethal predator management activities implemented by land managers to increase reproductive outcomes and recovery of at-risk beach species^57–59^. If it can be shown that allowing leashed dogs in protected areas has a sizable benefit to at-risk species vis-a-vis suppressed predator activity, it may warrant consideration of conditions where dog activity might provide some advantage. Additional study of the impacts of leashed vs. unleashed dogs on at-risk wildlife may shed information on whether leashed dogs can provide a window of opportunity for enhancing management of coastal species of conservation concern. Better understanding of the thresholds of dog disturbance may inform those situations where sufficient buffers can be provided to allow some dog activity while still minimizing their direct negative impacts.

From a broader ecological perspective, sandy beach ecosystems can be described as transition zones where massive amounts of organic matter and nutrients exchange freely across the marine to terrestrial interface. As such, sandy beaches are hotspots of biodiversity, drawing species from adjoining ecosystems and concentrating them spatially within a relatively narrow landscape extent^29^. This biodiversity results in high rates of ecological function, and due to their direct and indirect effects on resident wildlife communities, domestic dogs (even leashed ones) interrupt the connectedness of the beach environment and the species it supports.

## Methods

### Study area and data collection

We sampled the scavenger guild in September and October of 2020 at 63 sites located along ~ 250 km of ocean-exposed coastline in New Jersey, USA. The study region extended from Gateway National Recreation Area—Sandy Hook Unit (40.44098745004098, −73.98901519050182) in the north to Cape May Borough (38.931442, −74.959019) in the south (Fig. 2). Temperature and rainfall in coastal New Jersey average 19.4 °C and 8.1 cm, respectively. Sites within the study region included both mainland and barrier beaches that varied in size, shape and flora vegetative characteristics. Beaches consist of primary dunes dominated by American beach grass (*Ammophila breviligulata*), secondary dunes consisting of northern bayberry (*Myrica pensylvanica*) and other woody shrubs, and maritime forest dominated by eastern red cedar (*Juniperus virginiana*). The human footprint of each site also varied from unpopulated and undeveloped beaches (i.e. national wildlife refuges) to heavily urbanized, highly populated, resort towns. Presumably all dogs within the study area can be considered owned and cared for by a human companion. Local dog laws throughout the study region range from prohibited to allowed. Although leash laws are in effect, owners often exercise their dogs off-leash on beaches within the study area.

At each site we deployed a single infrared remote-triggered camera (Reconyx HP2X Hyperfire 2, Reconyx, Inc., Holmen, WI, USA) on the upper beach at or just above the spring high water mark for a period of 24 h. Previous work has demonstrated that a 24-h survey period is sufficient to capture the dynamics of scavenger communities in coastal landscapes^60,61^. Camera trap locations were at least 2 km apart to avoid detections of the same individual on multiple camera traps. They were placed away from obvious wildlife trails or other features likely to influence the unbiased detection of scavengers^62,63^. We mounted cameras on wooden posts at a height of 75 cm, with the camera face directed toward the ground at ~ 45^ o^ ^64^. We set motion triggers to high sensitivity and programmed cameras to capture a burst of 3 photos taken at 1-s intervals. To improve accuracy of species identification, we followed each photo burst with a single 10-s video. We baited each camera station twice during the 24-h survey period, initially between the beginning of civil twilight and sunrise (0 h), and again between sunset and the end of civil twilight (12 h), to capture both diurnal and nocturnal scavengers within the guild (Bingham 2018, Schlacher 2019). At each baiting event, we placed 3 freshly thawed Atlantic menhaden (*Brevoortia tyrannus*) carcasses directly within the field of view of each camera after weighing them with a digital scale (total necromass: 762.5 ± 143.6 g SD). At the 12- and 24-h survey marks, we weighed the remaining carrion.

In the lab we recorded the number of scavenger species captured at each camera location. An animal was considered a scavenger if it was observed investigating, consuming, or removing carrion from the camera station. Because individuals were not marked and display nontrivial differences in their dispersion patterns^65,66^, we recorded presence or absence of species rather abundance. We acknowledge that crows captured on cameras could have been either American crows (*Corvus brachyrhynchos*) or fish crows (*C. ossifragus*), as these species can only reliably be distinguished by their calls. Therefore, we considered all crows at the genus level for all reporting and analyses.

### Contrasting scavenging on beaches with and without dogs

We compared species composition of scavenger communities between beaches with and without dogs with permutational multivariate analysis of variance (PERMANOVA) based on Bray–Curtis dissimilarity matrices calculated from the species presence/absence at camera stations^67^. We used a SIMPER analysis to calculate the contribution of individual species to community-level differences between ‘dog’ and ‘no-dog’ locations during diurnal and nocturnal deployments. Community composition analyses were performed using the “adonis2” and “simper” functions in the ‘vegan’ R package^68,69^. We contrasted three metrics of the scavenging function: (1) number of species visiting each camera station; (2) time to carcass detection (number of minutes elapsed after carcass deployment until the first scavenger is detected at a camera station); and 3) the proportion of carrion consumed. We then used one-way analysis of variance (ANOVA) to investigate separately the potential differences in scavenging metrics between beaches where dogs were and were not observed during the day.

### Factors influencing scavenging function on ocean-exposed beaches

Prior to model development, we found not statistical correlations among our selected predictor variables. We developed 33 a priori candidate models (Supplemental Information–Table S1) designed to explicitly test the effect of owned dogs on our 3 predictor variables relative to other established factors^61,70^. Each candidate model set was also compared to a global model, which included all predictor variables measured. Dogs were considered present if at least one dog was observed on the camera during deployment. While dogs could be generally individually identified, our remote camera approach provided no indication of dog behavior or time spent at the site. A single, unleashed dog remaining on site for multiple hours may have a greater effect than multiple leashed dogs walking briefly through the site. Therefore, we conservatively modeled dog presence as a categorical variable. Predictor variables included time of carcass deployment (day or night), total area of beach within a 500-m radius of the camera station, total area (in hectares) of natural land cover (i.e. vegetation, sand), distance to development (Euclidean distance in meters from the camera location to the nearest manmade land cover class), and human population density (New Jersey Department of Labor and Workforce Development). We also classified beaches based on whether they were managed as natural ecosystems (limited site-level management) or recreational venues (i.e. daily groomin, presence of garbage receptables, etc.) Landscape-level predictors were quantified using New Jersey Land Use/Land Cover 2015 Update, Edition 20190128 obtained from the NJ Department of Environmental Protection (NJDEP) and ArcGIS Pro 2.9 software (ESRI). Population densities for study sites were derived from the New Jersey Department of Labor and Workforce Development census data (https://nj.gov/health/fhs/primarycare/documents/Rural%20NJ%20density2015-revised%20municpalities.pdf). All models included site as a random effect.

We modeled the number of visiting species and the proportion of carrion consumed using generalized linear mixed effect models (GLMMs) with either a Poisson or binomial distribution as dictated by the data using the package lme4 (Bates et al. 2007). To evaluate time to carcass detection, we used a two-part mixed effects hurdle model approach using the package glmmTMB (Brooks et al. 2017), whereby a logistic model first considers whether carrion was detected and a count-based GLMM (following a negative binomial distribution with a log-link function) then models time to carcass detection. In this way, we account for camera traps where carrion was never detected^71^. For each model set, we used small-sample corrected Akaike’s Information Criterion (AIC_c_) to select candidate models^72^. To reduce model selection bias and uncertainty, we averaged all models returning a $\Delta$AIC_c_ < 2 using the packages bbmle and MuMin^73,74^ and calculated parameter estimates based on weighted averages of the parameter estimates included in the top models^72^. We performed all analyses in R and used the dplyr and ggplot2 packages for all data manipulations and visualizations^75,76^.

## Supplementary Information

Supplementary Information.

## Data Availability

Data are available from the corresponding author, Dr. Brooke Maslo, by request.

## References

[1] Hughes J, Macdonald DW (2013). A review of the interactions between free-roaming domestic dogs and wildlife. Biol. Cons..

[2] Doherty TS (2017). The global impacts of domestic dogs on threatened vertebrates. Biol. Cons..

[3] Young JK, Olson KA, Reading RP, Amgalanbaatar S, Berger J (2011). Is wildlife going to the dogs? Impacts of feral and free-roaming dogs on wildlife populations. Bioscience.

[4] Ritchie, E. G., Dickman, C. R., Letnic, M., Vanak, A. T. & Gommper, M. Dogs as predators and trophic regulators. *Free-ranging dogs and wildlife conservation*, 55–68 (2014).

[5] Gompper, M. E. In *Free-ranging dogs and wildlife conservation*, Oxford University Press (2014).

[6] Somaweera R, Webb JK, Shine R (2011). It’sa dog-eat-croc world: Dingo predation on the nests of freshwater crocodiles in tropical Australia. Ecol. Res..

[7] Weston, M. A. & Stankowich, T. In *Free-Ranging Dogs and Wildlife Conservation. ME Gompper (ed.)* (ed Matthew E Gompper) Ch. 4, 94–113 (Oxford University Press, 2013).

[8] Zapata-Ríos G, Branch LC (2016). Altered activity patterns and reduced abundance of native mammals in sites with feral dogs in the high Andes. Biol. Cons..

[9] Donadio E, Buskirk SW (2006). Diet, morphology, and interspecific killing in Carnivora. Am. Nat..

[10] Gingold G, Yom-Tov Y, Kronfeld-Schor N, Geffen E (2009). Effect of guard dogs on the behavior and reproduction of gazelles in cattle enclosures on the Golan Heights. Anim. Conserv..

[11] Fernández-Juricic E, Tellería JL (2000). Effects of human disturbance on spatial and temporal feeding patterns of Blackbird Turdus merula in urban parks in Madrid, Spain. Bird Study.

[12] Vanak AT, Gompper ME (2009). Dogs Canis familiaris as carnivores: Their role and function in intraguild competition. Mammal Rev..

[13] Silva-Rodríguez EA, Sieving KE (2012). Domestic dogs shape the landscape-scale distribution of a threatened forest ungulate. Biol. Cons..

[14] Banks PB, Bryant JV (2007). Four-legged friend or foe? Dog walking displaces native birds from natural areas. Biol. Let..

[15] Langston R, Liley D, Murison G, Woodfield E, Clarke R (2007). What effects do walkers and dogs have on the distribution and productivity of breeding European Nightjar Caprimulgus europaeus?. Ibis.

[16] Lenth BE, Knight RL, Brennan ME (2008). The effects of dogs on wildlife communities. Nat. Areas J..

[17] Weston, M. A. & Stankowich, T. Dogs as agents of disturbance. *Free-Ranging Dogs and Wildlife Conservation. ME Gompper (ed.)*, 94–113 (2013).

[18] Letnic M, Ritchie EG, Dickman CR (2012). Top predators as biodiversity regulators: The dingo Canis lupus dingo as a case study. Biol. Rev..

[19] Maguire, G. S., Miller, K. K. & Weston, M. A. In *Impacts of Invasive Species on Coastal Environments* 397–412 (Springer, 2019).

[20] Cardinale BJ (2012). Biodiversity loss and its impact on humanity. Nature.

[21] Delgado-Baquerizo M (2016). Microbial diversity drives multifunctionality in terrestrial ecosystems. Nat. Commun..

[22] Rodriguez LF (2006). Can invasive species facilitate native species? Evidence of how, when, and why these impacts occur. Biol. Invasions.

[23] Rosenfeld JS (2002). Functional redundancy in ecology and conservation. Oikos.

[24] Díaz S, Fargione J, Chapin FS, Tilman D (2006). Biodiversity loss threatens human well-being. PLoS Biol.

[25] Hooper DU (2012). A global synthesis reveals biodiversity loss as a major driver of ecosystem change. Nature.

[26] Barbier EB (2011). The value of estuarine and coastal ecosystem services. Ecol. Monogr..

[27] Nel R (2014). The status of sandy beach science: Past trends, progress, and possible futures. Estuar. Coast. Shelf Sci..

[28] Schlacher TA (2015). Golden opportunities: A horizon scan to expand sandy beach ecology. Estuar. Coast. Shelf Sci..

[29] Schlacher TA (2019). Key ecological function peaks at the land–ocean transition zone when vertebrate scavengers concentrate on ocean beaches. Ecosystems.

[30] Lockwood, J. L. & Maslo, B. In *Coastal Convervation* (eds Brooke Maslo & JL Lockwood) 1–10 (Cambridge University Press, 2014).

[31] Morin DJ, Lesmeister DB, Nielsen CK, Schauber EM (2018). The truth about cats and dogs: Landscape composition and human occupation mediate the distribution and potential impact of non-native carnivores. Glob. Ecol. Conserv..

[32] Cortés EI, Navedo JG, Silva-Rodríguez EA (2021). Widespread presence of domestic dogs on sandy beaches of Southern Chile. Animals.

[33] Burger J, Jeitner C, Clark K, Niles LJ (2004). The effect of human activities on migrant shorebirds: Successful adaptive management. Environ. Conserv..

[34] Dowling B, Weston MA (1999). Managing a breeding population of the Hooded Plover Thinornis rubricollis in a high-use recreational environment. Bird Conserv. Int..

[35] Vanak AT, Gompper ME (2010). Interference competition at the landscape level: The effect of free-ranging dogs on a native mesocarnivore. J. Appl. Ecol..

[36] Marzluff, J. M., McGowan, K. J., Donnelly, R. & Knight, R. L. In *Avian ecology and conservation in an urbanizing world* 331–363 (Springer, 2001).

[37] Handler A, Lonsdorf EV, Ardia DR (2020). Evidence for red fox (Vulpes vulpes) exploitation of anthropogenic food sources along an urbanization gradient using stable isotope analysis. Can. J. Zool..

[38] Prange S, Gehrt SD, Wiggers EP (2003). Demographic factors contributing to high raccoon densities in urban landscapes. The J. Wildlife Manag..

[39] Méndez A (2020). Adapting to urban ecosystems: unravelling the foraging ecology of an opportunistic predator living in cities. Urban Ecosyst..

[40] Rees J, Webb J, Crowther M, Letnic M (2015). Carrion subsidies provided by fishermen increase predation of beach-nesting bird nests by facultative scavengers. Anim. Conserv..

[41] Kimber O (2020). The fox and the beach: Coastal landscape topography and urbanisation predict the distribution of carnivores at the edge of the sea. Glob. Ecol. Conserv..

[42] Ruxton GD, Houston DC (2004). Obligate vertebrate scavengers must be large soaring fliers. J. Theor. Biol..

[43] Cortés-Avizanda A, Jovani R, Donázar JA, Grimm V (2014). Bird sky networks: How do avian scavengers use social information to find carrion?. Ecology.

[44] Harel R, Spiegel O, Getz WM, Nathan R (2017). Social foraging and individual consistency in following behaviour: Testing the information centre hypothesis in free-ranging vultures. Proc. Royal Soc. B: Biol. Sci..

[45] Soulsbury CD, Iossa G, Baker PJ, White PC, Harris S (2011). Behavioral and spatial analysis of extraterritorial movements in red foxes (Vulpes vulpes). J. Mammal..

[46] Johnson CN, VanDerWal J (2009). Evidence that dingoes limit abundance of a mesopredator in eastern Australian forests. J. Appl. Ecol..

[47] Polis GA, Anderson WB, Holt RD (1997). Toward an integration of landscape and food web ecology: The dynamics of spatially subsidized food webs. Ann. Rev. Ecol. Syst..

[48] Barton PS, Cunningham SA, Lindenmayer DB, Manning AD (2013). The role of carrion in maintaining biodiversity and ecological processes in terrestrial ecosystems. Oecologia.

[49] Schlacher TA, Strydom S, Connolly RM (2013). Multiple scavengers respond rapidly to pulsed carrion resources at the land–ocean interface. Acta Oecologica.

[50] Dunbrack TR, Dunbrack RL (2010). Why take your dog on a picnic: presence of a potential predator (Canis lupus familiaris) reverses the outcome of food competition between northwestern crows (Corvus caurinus) and glaucous-winged gulls (Larus glaucescens). Northwest. Nat..

[51] Jiménez J (2019). Restoring apex predators can reduce mesopredator abundances. Biol. Cons..

[52] Bhadra A (2016). The meat of the matter: A rule of thumb for scavenging dogs?. Ethol. Ecol. Evol..

[53] Turner KL, Abernethy EF, Conner LM, Rhodes OE, Beasley JC (2017). Abiotic and biotic factors modulate carrion fate and vertebrate scavenging communities. Ecology.

[54] Ogada D, Torchin M, Kinnaird M, Ezenwa V (2012). Effects of vulture declines on facultative scavengers and potential implications for mammalian disease transmission. Conserv. Biol..

[55] O’Bryan CJ (2018). The contribution of predators and scavengers to human well-being. Nat. Ecol. & Evol..

[56] Gómez-Serrano MÁ (2021). Four-legged foes: Dogs disturb nesting plovers more than people do on tourist beaches. Ibis.

[57] Stantial M, Cohen J, Darrah A, Farrell S, Maslo B (2021). The effect of top predator removal on the distribution of a mesocarnivore and nest survival of an endangered shorebird. Avian Conserv. Ecol..

[58] Mahon PS (2009). Targeted control of widespread exotic species for biodiversity conservation: The red fox (Vulpes vulpes) in New South Wales, Australia. Ecol. Manag. Restor..

[59] Colwell, M. A. In *The Population Ecology and Conservation of Charadrius Plovers* 127–147 (CRC Press, 2019).

[60] Huijbers CM (2015). Limited functional redundancy in vertebrate scavenger guilds fails to compensate for the loss of raptors from urbanized sandy beaches. Divers. Distrib..

[61] Huijbers CM, Schlacher TA, Schoeman DS, Weston MA, Connolly RM (2013). Urbanisation alters processing of marine carrion on sandy beaches. Landsc. Urban Plan..

[62] Meek P (2014). Recommended guiding principles for reporting on camera trapping research. Biodivers. Conserv..

[63] Kolowski JM, Forrester TD (2017). Camera trap placement and the potential for bias due to trails and other features. PLoS ONE.

[64] Burton AC (2015). Wildlife camera trapping: a review and recommendations for linking surveys to ecological processes. J. Appl. Ecol..

[65] Selva N, Fortuna MA (2007). The nested structure of a scavenger community. Proc. Royal Soc. B: Biol. Sci..

[66] Olson ZH, Beasley JC, Rhodes OE (2016). Carcass type affects local scavenger guilds more than habitat connectivity. PLoS ONE.

[67] Anderson MJ (2001). Permutation tests for univariate or multivariate analysis of variance and regression. Can. J. Fish. Aquat. Sci..

[68] Team, R. D. C. R: A language and environment for statistical computing. *R Foundation for statistical computing, Vienna, Austria* (2013).

[69] Dixon P (2003). VEGAN, a package of R functions for community ecology. J. Veg. Sci..

[70] Schlacher TA (2015). Conservation gone to the dogs: When canids rule the beach in small coastal reserves. Biodivers. Conserv..

[71] Lewin W-C, Freyhof J, Huckstorf V, Mehner T, Wolter C (2010). When no catches matter: Coping with zeros in environmental assessments. Ecol. Ind..

[72] Burnham, K. P. & Anderson, D. R. *Model selection and multimodel inference: a practical information-theoretic approach*. 488 (Springer Science & Business Media, 2002).

[73] Bolker, B. & Team, R. (R package version 0.9, 2010).

[74] Barton, K. & Barton, M. K. Package ‘mumin’. *Version* **1**, 439 (2015).

[75] Wickham, H., Francois, R., Henry, L. & Müller, K. dplyr: A grammar of data manipulation. R package version 0.4. 3. *R Found. Stat. Comput., Vienna*. https://CRAN. R-project. org/package= dplyr (2015).

[76] Wickham, H., Chang, W. & Wickham, M. H. Package ‘ggplot2’. *Create Elegant Data Visualisations Using the Grammar of Graphics. Version* **2**, 1–189 (2016).

