# Supplementary material for: Dogs suppress a pivotal function in the food webs of sandy beaches
Source: Sci Rep. 2022 Aug 18;12:14069. doi: 10.1038/s41598-022-18194-9 (PMC9388640; doi:10.1038/s41598-022-18194-9)
Supplement: Supplementary file 1 — Supplementary Information. [file 41598_2022_18194_MOESM1_ESM.pdf]

Supplemental Information

Dogs suppress a pivotal function in the food webs of sandy beaches

Brooke Maslo[1*], Robert Kwait[1], Christian Crosby[1], Price Holman[1], Isabelle Zoccolo[1], Kathleen Kerwin[1], Todd Pover[2] and Thomas A. Schlacher[3]

[1] Department of Ecology, Evolution and Natural Resources, Rutgers, The State University of New Jersey, New Jersey USA; rek89@scarletmail.rutgers.edu; peh53@scarletmail.rutgers.edu; isabelle.zoccolo@rutgers.edu; chc84@sebs.rutgers.edu; Kathleen.kerwin@rutgers.edu

[2] Conserve Wildlife Foundation of New Jersey, Princeton, New Jersey, USA; todd.pover@conservewildlifenj.org

[3] School of Science and Engineering, University of the Sunshine Coast, Maroochydore, Australia; tschlach@usc.edu.au

* Correspondence: brooke.maslo@rutgers.edu

Table S1. List of *a priori* candidate models used in regression analyses testing the effect of predictor variables on three scavenging function metrics: 1) number of species visiting each camera station; 2) time to carcass detection (number of minutes elapsed after carcass deployment until the first scavenger is detected at a camera station); and 3) the proportion of carrion consumed.

Model #	Predictor Variables
1	deployment period
2	dogs
3	site type
4	deployment period + beach area
5	deployment period*beach area
6	deployment period + beach area + population density + distance to development
7	deployment period + dogs
8	deployment period + population density
9	deployment period + site type
10	deployment period*site type
11	deployment period + dogs + distance to development + population density
12	deployment period + dogs + population density
13	deployment period*dogs
14	(deployment period*dogs) + distance to development + population density
15	(deployment period*dogs) + population density
16	deployment period + natural area cover + site type + beach area
17	deployment period*(natural area cover + beach area)
18	deployment period*(natural area cover + beach area + site type)
19	deployment period*(beach area + distance to development)
20	deployment period + site type + + distance to development
21	deployment period*(distance to development + population density)
22	deployment period*(distance to development + population density + beach area)
23	dogs + population density
24	dogs + population density + distance to development
25	dogs*site type
26	dogs + distance to development + population density + site type
27	beach area + distance to development + population density
28	distance to development + population density
29	distance to development + population density + site type
30	beach area + natural area cover
31	beach area + natural area cover + site type
32	site type*(beach area + population density + distance to development)
33	global model

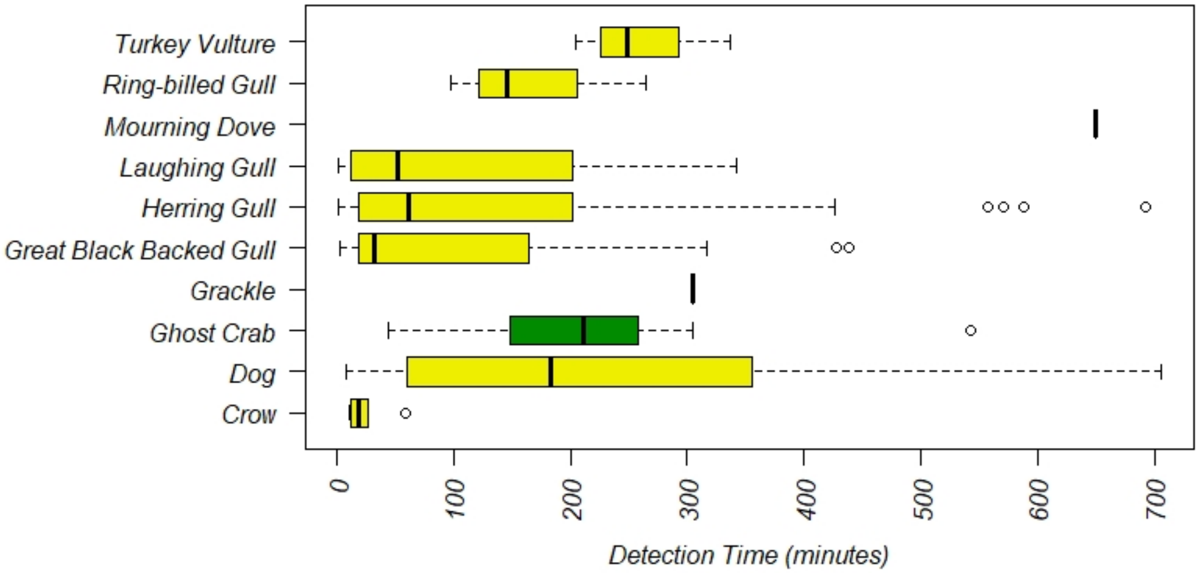

Figure S1. Box plots indicating the time to carcass detection for diurnal scavengers on in sandy beach ecosystems. Atlantic ghost crabs (green) were observed during both day and night deployments.

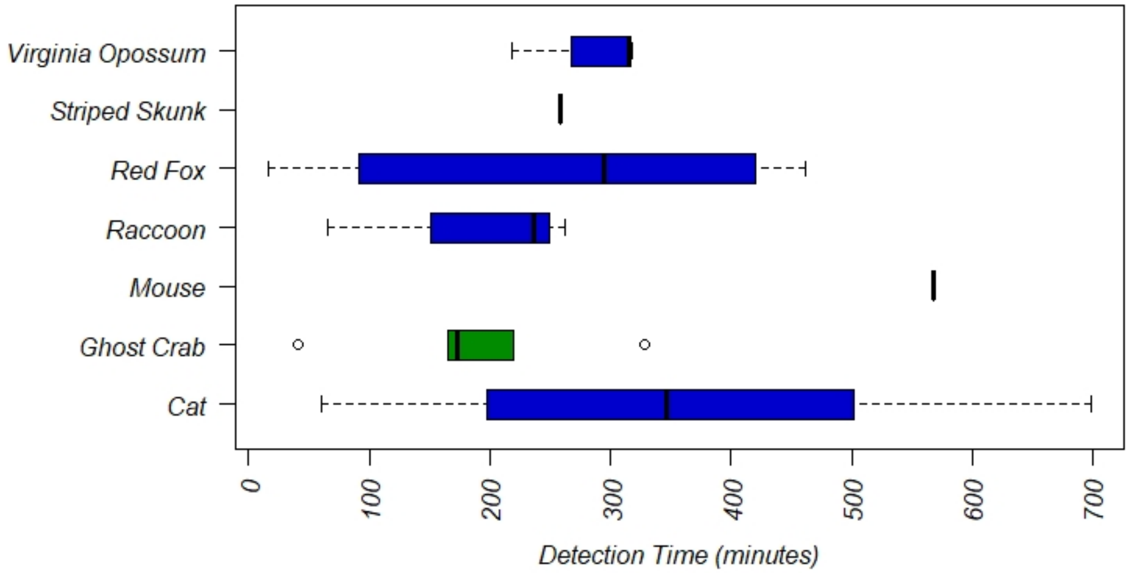

Figure S2. Box plots indicating the time to carcass detection for nocturnal scavengers on in sandy beach ecosystems. Atlantic ghost crabs (green) were observed during both day and night deployments.
